# The Neuroanatomy of Induced Pluripotent Stem Cells: In Vitro Models of Subcortical Nuclei in Neurodegenerative Disorders

**DOI:** 10.3390/cimb46090607

**Published:** 2024-09-14

**Authors:** Alessandro Galgani, Marco Scotto, Filippo S. Giorgi

**Affiliations:** 1 Department of Translational Research and of New Surgical and Medical Technologies, University of Pisa, 56126 Pisa, Italy; 2 Istituto Italiano di Tecnologia, 16163 Genova, Italy; 3 IRCCS Stella Maris Foundation, 56128 Pisa, Italy

**Keywords:** noradrenaline, dopamine, acetylcholine, serotonin, Alzheimer’s disease, Parkinson’s disease, neurodegeneration, induced pluripotent stem cells

## Abstract

Neuromodulatory subcortical systems (NSSs) are monoaminergic and cholinergic neuronal groups that are markedly and precociously involved in the pathogenesis of many neurodegenerative disorders (NDDs), including Parkinson’s and Alzheimer’s diseases. In humans, although many tools have been developed to infer information on these nuclei, encompassing neuroimaging and neurophysiological methods, a detailed and specific direct evaluation of their cellular features in vivo has been difficult to obtain until recent years. The development of induced pluripotent stem cell (iPSC) models has allowed research to deeply delve into the cellular and molecular biology of NSS neurons. In fact, iPSCs can be produced easily and non-invasively from patients’ fibroblasts or circulating blood monocytes, by de-differentiating those cells using specific protocols, and then be re-differentiated towards neural phenotypes, which may reproduce the specific features of the correspondent brain neurons (including NSS ones) from the same patient. In this review, we summarized findings obtained in the field of NDDs using iPSCs, with the aim to understand how reliably these might represent in vitro models of NSS. We found that most of the current literature in the field of iPSCs and NSSs in NDDs has focused on midbrain dopaminergic neurons in Parkinson’s disease, providing interesting results on cellular pathophysiology and even leading to the first human autologous transplantation. Differentiation protocols for noradrenergic, cholinergic, and serotoninergic neurons have also been recently defined and published. Thus, it might be expected that in the near future, this approach could extend to other NSSs and other NDDs.

## 1. Introduction

Neurodegenerative disorders (NDDs) are among the most pressing medical issues of our time. The global burden of Alzheimer’s disease (AD) and Parkinson’s disease (PD) increases every year, and effective disease-modifying therapies are yet to be developed and introduced into clinical practice [1,2]. At the same time, a precision medicine approach is essential, as the same symptoms and degeneration of similar brain nuclei in patients with the disease may be related to different pathogenetic mechanisms at an individual level. A constant key element of NDD pathogenesis is the involvement of the neuromodulatory subcortical systems (NSSs) from the early stages of the diseases [3]. NSSs are a group of subcortical nuclei distributed along the brainstem and the most phylogenetically ancient part of the telencephalon [4]. They play crucial roles in regulating central nervous system (CNS) homeostasis and modulating its activities both at a microscale and macroscale level. In PD, the degeneration of one of these NSSs, the pars compacta of the mesencephalic nucleus substantia nigra (SNpc), is the main driver of the disease symptomatology [5]. In AD, the disruption of the cholinergic (Ach) innervation of the limbic cortex, arising from the nucleus basalis of Meynert (NBM) and the medial septal nucleus (MSN), plays a key role in the core symptoms of the disease, i.e., memory impairment [6,7]. Interestingly, in both disorders, the early impairment of the pontine noradrenergic (NA) locus coeruleus (LC) (which represents one of the main NSSs, due to the particularly widespread innervation of the whole brain by its axons, see Section 2.2) has been hypothesized to play a significant pathogenic role by disrupting precociously neuronal homeostasis [8,9,10,11]. Furthermore, sleep disturbances and mood alterations, common accompanying symptoms in NDDs, have been linked to the impairment of orexinergic neurons in the lateral hypothalamus (LHA) and serotoninergic (5HT) dorsal raphe nucleus (DRN), respectively [12,13,14].

In the last decades, our understanding of NDDs’ pathogenesis and pathophysiology has remarkably improved, although their etiology remains largely unclear. Arguably, the bottleneck may be the difficulty in directly accessing CNS functioning in vivo in patients. This constraint limits research on NDDs in humans to indirect in vivo tools, such as neuroimaging and neurophysiological measurements, or to static morphological assessments post-mortem of specific brain areas/networks. Concerning NSSs, many neuroimaging tools have been developed to evaluate the integrity of these nuclei in vivo; some have stably entered clinical practice (e.g., Fluoro-DOPA positron emission tomography or DATscan single photon emission computed tomography) [5], while others are limited to research purposes (e.g., neuromelanin-sensitive magnetic resonance imaging for SNpc and LC) [15,16].

In this context, a promising experimental approach that may open a direct window on human NSSs is the development of induced pluripotent stem cell (iPSC) models [17,18,19,20,21,22]. Fully differentiated mesenchymal cells (such as fibroblasts or circulating blood monocytes) [18,23] can be de-differentiated from iPSCs using specific protocols, and then re-programmed towards neural phenotypes [17], and even further to specific neuronal population phenotypes, such as dopaminergic (DA) or NA cells of the brainstem. This potentially allows researchers to perform experimental studies directly on human cells belonging to one of the NSSs’ nuclei, assessing in vivo cellular and molecular pathways that might play a pathogenetic role and/or the effects of specific drugs on those [19,20,21,22]. Furthermore, since iPSCs can be developed from the very same cells of the patients, they provide the opportunity to assess any differences in cell physiology and metabolism and to associate them with the characteristics of the donor [19,20,21,22].

In this review, we will provide a global perspective on the use of iPSCs as a model for studying NSSs, with the following dual purpose: (a) to assess whether the current state-of-the-art differentiation protocols offer the potential to “replicate” all of the main NSSs (specifically NA LC, DA SNpc, Ach NBM, and 5HT DRN) and (b) to summarize the primary experimental findings obtained thus far, from both pathophysiological and therapeutic viewpoints, in the context of NDD research. To facilitate a comprehensive understanding of our findings, we will begin by briefly introducing the NSSs from anatomical and functional perspectives.

## 2. The Neuromodulatory Subcortical Systems: Elements of Functional Anatomy

From an anatomical point of view, NSSs belong to the so-called “isodendritic core”, which includes several clusters of neurons sparsely distributed along the brainstem and basal telencephalon and classified based on their main neurotransmitter [24]. The key and common denominator feature of NSSs is represented by the isodendritic neuron, characterized by vast dendritic arborization and axonal branching. This allows it to convey a large amount of input stimuli and to reach broad areas of the CNS with its output signal [25]. This specific cytoarchitecture is the morphological counterpart of the main physiological function of the isodendritic core, which is to regulate the activity of the CNS based on the external and internal environment, and, in particular, to modulate the sleep/wake cycle and the state of vigilance [26].

Within this functional framework, even though they show different cytoarchitecture or topography, other neuronal clusters are also classically included in the definition of NSSs, such as the Ach nuclei of the basal forebrain [27] or the orexinergic neurons of the LHA [12]. Additionally, NSSs play specific roles depending on their main neurotransmitter, influencing specific cellular metabolic pathways, neural tissue homeostasis, and neuronal networks.

For the purposes of this review, we summarize the NSSs’ main features according to histochemical classification (based on their principal neurotransmitter) [28,29,30], reporting the nuclei that are more relevant for human neurobiology, in a caudo-rostral order (see also Table 1 for a schematic description). 

### 2.1. The Raphe Nuclei and the Serotoninergic System 

The raphe nuclei are a group of heterogeneous clusters of neurons distributed along the median line of the brainstem, from the medulla oblongata to the midbrain, mainly characterized by the synthesis and release of 5HT. Classified by Dahlstrom and Fuxe into nine different clusters (B1–B9) [28,29], the highest density of 5HT neurons is found in the DRN (B8–B9), which is located at the junction between the pons and the midbrain [28,29]. The DRN projects to several structures of the CNS, including other nuclei of the brainstem, the hypothalamus, the amygdala, the hippocampus, and the cortex [31,32]. Through this wide innervation pattern, 5HT plays pivotal roles in the sleep/wake cycle (promoting wakefulness), pain modulation (projecting onto the periaqueductal gray), vegetative functions, and regulation of mood and affective behavior [31,32,33,34].

Functional alterations and structural disruption of the 5HT NSS can be detected in the early stages of many degenerative diseases. Furthermore, both PD and AD patients often experience prodromal symptoms, including mood and sleep disturbances, that might be associated with 5HT NSS impairment [35].

### 2.2. The Locus Coeruleus and the Noradrenergic System

Although seven clusters of NA neurons can be detected along the brainstem, the main hub of brain NA system is by far the pontine nucleus LC (A6, according to the Dahlstrom and Fuxe classification), which is placed just below the floor of the fourth ventricle [29,36]. The LC is a tiny tube-shaped nucleus that, through its projections, reaches virtually all areas of the CNS. On a macroscale level, it plays crucial roles in the sleep/wake cycle, acting as a wakefulness and alertness-promoting nucleus, modulating fear and anxiety, and promoting attention [37,38,39]. At the cellular level, NA exerts a neuroprotective effect by regulating neurovascular coupling, promoting blood–brain barrier integrity, modulating microglial activity, and inducing neural growth factor production [40,41,42,43].

The involvement of LC in NDDs has gained increasing attention in recent years. In AD, the LC may be the very first brain structure to show detectable pathological alterations, such as hyperphosphorylated tau accumulation, which has been shown to occur even decades before the clinical onset of the disease [9,44]. In PD and other parkinsonisms, LC degeneration also occurs early and is associated with several non-motor symptoms [45,46]. Furthermore, the loss of its physiological neuroprotective effects is now considered one of the possible pathogenic mechanisms contributing to the progression of these disorders, as the loss of NA innervation has been linked to increased neuroinflammation [47,48], pathological protein accumulation [49], and neurovascular alterations [50].

### 2.3. The Dopaminergic Nuclei: The Substantia Nigra and the Ventral Tegmental Area

The DA NSS is mainly represented by two nuclei: the SNpc and the ventral tegmental area (VTA), which are both placed in the midbrain close to each other. The DA neurons of the SNpc project to the striatum (nigrostriatal pathway), engage in a complex interplay with the motor cortex, thalamus, and subthalamic nucleus of Lewis to regulate movement planning and execution [4,29]. The VTA projects to both limbic structures, such as the amygdala, hippocampus, and nucleus accumbens (mesolimbic pathway), and prefrontal/frontal cortices (mesocortical pathway). Through its mesolimbic connections, the VTA participates in the reward process and reinforced learning, while the mesocortical pathway allows the modulation of cognitive functions, such as executive function and planning [51,52,53,54].

The degeneration of the SNpc is the key pathological feature of PD (together with LC neuronal loss) and, to varying extents, also occurs in other parkinsonisms. The loss of nigrostriatal DA is key in the classical motor symptoms of PD, such as tremors, bradykinesia, and rigidity [5,45]. VTA impairment occurs in PD but can also be detected in AD patients and is involved in psychiatric and behavioral alterations, such as psychosis, hallucinations, and apathy [55,56].

### 2.4. The Cholinergic Nuclei

The anatomical distribution of the Ach nuclei along the encephalon is more heterogeneous compared with the NSSs described above. According to Mesulam’s classification, six main clusters of Ach neurons can be found in the human CNS [27]. Two of them, the pedunculopontine nucleus (Ch5) (PPN) and the lateral tegmental nucleus (Ch6) (LTN), are located at the junction between the pons and the midbrain, laterally to the LC. They mainly participate in the regulation of the sleep/wake cycle, functioning as wake-promoting nuclei [27]. The other four Ach nuclei are placed in the basal forebrain and are the NBM (Ch1), the MSN (Ch2), and the horizontal (Ch3) and vertical (Ch4) limbs of the diagonal band of Broca (hDBB and vDBB, respectively). Despite their position, they are considered as the most rostral extension of the isodendritic core. The hDBB projects to the olfactory cortex, the vDBB and the NSM to the hippocampus, and the NBM to the isocortex [27,30,57]. The Ach nuclei of the basal forebrain play a key role in cortical activation, promoting the desynchronization of neuronal activity and facilitating memory and learning [58,59]. At the cellular level, Ach also regulates neurovascular coupling and exerts anti-inflammatory effects. The degeneration of these nuclei, particularly the NBM, occurs in AD and is associated with its key symptom, i.e., memory impairments [6,7]. Also, in PD and Lewy body dementia, the involvement of this NSS is strictly linked to cognitive impairment [60,61]. 

## 3. Methodology of the Literature Search

We performed a literature search on PubMed.com using “iPSC”, “induced pluripotent stem cells”, “neurons”, “noradrenaline”, “dopamine”, “acetylcholine”, and “serotonin” as key words. We found 1618 records, and 1208 were excluded upon title analysis because they were out of topic. We also excluded 278 papers after abstract analysis and 34 more after text reading. Ninety-eight studies were finally included. While, for the first part of this review, all studies describing NSS iPSCs were considered suitable, for its second part, dedicated to the description of pathophysiological and pharmacological findings, only papers concerning NDDs and related symptoms were considered. 

## 4. NSS iPSCs: Differentiation Protocols

### 4.1. Reprogramming of Somatic Cells

Human iPSC derivation methods encompass various strategies to reprogram somatic cells into a pluripotent state. In order to obtain neural precursors from somatic cells, mainly fibroblasts [62,63,64,65,66,67,68,69,70,71,72,73,74,75,76,77,78,79,80,81,82,83,84] and peripheral blood mononuclear cells (PBMCs) [85,86,87,88,89], various reprogramming methods have been used over the years to produce iPSCs. One of the first reprogramming approaches was the original method developed by Yamanaka et al. (2006), which involved reprogramming with retroviral vectors by infecting differentiated cells with retroviruses carrying key reprogramming genes, encoding for the transcription factors POU domain class 5 transcription factor 1 (OCT3/4), the sex-determining region Y-box2 (SOX2), Kruppel-like factor 4 (KLF4), and myelocytomatosis oncogene (c-MYC) [90]. While this approach has been successfully employed [72,77,78,79,81,83,91], further strategies for delivering reprogramming factors into somatic cells have been explored to obtain neural precursors. Lentiviral vectors have been used as a promising alternative [67,69,71,86,92,93,94], offering significant improvements in safety and efficiency compared with retroviral vectors [95,96]. However, both retroviral and lentiviral vectors share critical limitations due to the integration of reprogramming genes into the cellular genome; for this reason, alternative reprogramming methods that do not depend on integrating viral vectors are used. Among these, Sendai virus has been widely used [64,65,66,68,75,76,97,98], since its genome does not integrate into the host cell genome, thus avoiding insertional mutagenesis issues, and is not persistent during the post-reprogramming phases. Episomal vectors have also been used [62,63,70,85,87,88,89,92], although with lower reprogramming efficiency compared with integrating viral vectors [99].

### 4.2. Induction of Neural Precursors

The initial step of neural induction of iPSC is common for cells that are destined to acquire DA, NA, 5HT, and Ach fate and is based on the SMAD inhibition approach [62,63,64,66,67,68,69,71,72,73,74,76,77,81,87,88,89,91,92,93,94,97,98,100,101,102,103,104,105,106,107,108]. This step allows the generation of neural progenitors with forebrain identity [72,109], using small molecules that suppress transforming growth factor (TGF)-β pathways such as SB431542 [110] and the inhibitors or antagonists of bone morphogenetic proteins (BMPs) LDN193189, dorsomorphin, and noggin [111]. After forebrain induction, in order to generate progenitors with forebrain, midbrain, and hindbrain identity, the modulation of the sonic hedgehog (SHH) and WNT signaling pathways is essential [72,109]. Modulation of the SHH pathway is frequently performed using the same SHH or agonists, including purmorphamine and fibroblast growth factor 8 (FGF8) [62,63,64,65,66,67,68,69,71,72,74,76,77,81,82,83,84,87,88,89,91,92,93,94,97,98,101,102,103,104,105,106,107,108,109,112], and is used in 5HT and Ach neuron phenotype induction, after TGF-β and BMP inhibition, to induce the ventralization of hindbrain precursors [65,72,89,102,105]. For activation of the WNT pathway, the small compound CHIR99021, a potent inhibitor of GSK3b, is used in many protocols to promote the neural induction of progenitors of a variety of cell types, including DA, 5HT, and NA neurons [62,63,65,66,67,68,69,71,72,73,74,87,88,89,91,92,94,97,98,100,104,106,107,109]. During 5HT neural induction, in order to promote hindbrain precursor generation, rather than CHIR99021, Valiulahi et al. (2021) used retinoic acid, which, through the modulation of *HOX* family genes, controls hindbrain activation in a concentration-dependent manner [113]. In methods aiming at DA neural induction, the activation of the WNT pathway with CHIR99021 is widely used in combination with SHH or the aforementioned agonists [62,63,66,67,69,71,74,87,88,91,92,94,97,98,104,106,107,109], and promotes the expression of transcription factors Foxa2 and Lmx1a, which are involved in the midbrain specification of neural progenitors [114,115]. In this process, WNT signaling plays a crucial role compared with the SHH pathway. Indeed, it has been observed that the administration of SHH or agonists alone, in the absence of WNT activators, leads to a low expression of Foxa2, without any effects on Lmx1a [109]. In order to obtain NA neural progenitors, Tao et al. employed the WNT agonist CHIR99021 during the early phase of neural induction, following SMAD inhibition, to achieve the expression of midbrain differentiation-specific factors. Subsequently, by using different concentrations of the same molecule, they induced neural precursors towards a hindbrain identity [100].

### 4.3. Dopaminergic Differentiation 

Following the neural induction stage, in order to induce DA neuronal phenotypes, precursor cells are cultured in a neural maturation medium supplemented with specific growth factors and other compounds. Among these, commonly used factors are brain-derived neurotrophic factor (BDNF), glial-derived neurotrophic factor (GDNF), transforming growth factor β3 (TGFβ3), dibutyryl cyclic AMP (dbcAMP), N-[N-(3,5-difluorophenyl acetyl)-L-alanyl]-S-phenylglycine t-butyl ester (DAPT), and ascorbic acid (AA) [62,63,64,66,67,68,69,71,74,76,77,81,82,83,84,87,88,91,92,93,94,97,98,101,103,104,106,107,108,109,112,116]. In particular, the neurotrophic factors GDNF and BDNF are crucial for neuronal maturation by promoting the survival and differentiation of neural progenitors [117]. Similarly, TGFβ3 is thought to be involved in DA neuron survival [118] and functionally interacts with the BDNF and GDNF signaling pathways [119,120]. Omitting TGFβ3 from the neural maturation protocol during phenotype characterization results in the decreased expression of DA markers TH and FOXA2, alongside an increase in the astroglial genesis marker S100B [117]. Interestingly, dbcAMP, a cell-permeable cAMP analog, exhibits a differential effect on DA differentiation compared with the above-described growth factors. Although it has not clearly demonstrated its capability to promote neuronal survival, dbcAMP treatment during DA precursor maturation has been shown to significantly enhance the expression of TH in stem cell-derived neurons [117,121]. Finally, AA is frequently used in DA maturation protocols due to its epigenetic properties on modulating expression of key DA development genes such as FOXA2, LMX1A, and NURR1 through histone and DNA demethylation [122].

### 4.4. Noradrenergic Differentiation 

To the best of our knowledge, the differentiation of iPSCs to NA neurons has been described in only one recent paper until now. Following the acquisition of hindbrain identity through the modulation of the WNT signaling pathway, Tao et al. in 2023 [99] employed Activin A to induce neural progenitors towards an NA phenotype. Activin A, a member of the TGFβ superfamily, promotes the expression of genes crucial for NA fate, including Ascl1, Phox2a, and Phox2b, which exhibit partially overlapping functionalities [123,124]. The authors observed that administering Activin A at varying doses to neurons with rostral hindbrain identity resulted in early activation of Ascl1 at lower doses, and delayed activation of Phox2b at higher doses. In this context, aiming to enhance the expression of genes considered to be essential for acquiring NA fate, cyclopamine, an inhibitor of the SHH signaling pathway, was used. However, this treatment led to a reduction in the activation of genes necessary for inducing NA fate and a concomitant increase in the activation of genes that promote dorsalization of neural progenitors. Conversely, the administration of DMH1, an antagonist of the BMP signaling pathway, induced an upregulation of genes required for NA development, without causing significant dorsalizing effects. Finally, following the acquisition of NA fate, the growth factors BDNF, GDNF, and TGFβ and the compounds AA and cAMP were employed to facilitate subsequent neuronal maturation.

### 4.5. Serotoninergic Differentiation 

To generate 5HT neurons, it is crucial to establish hindbrain identity in neural precursors during the neural induction phase [65,72,73,89,102]. Protocols involving treatments with SHH and fibroblast growth factor 4 (FGF4) have been developed to induce the ventralization of hindbrain identity precursors [65,72]. Supplementation with FGF4 as an additional ventralization enhances the expression of transcription factor FOXA2 in synergy with SHH activation [65,72], and, in this context, FOXA2 is considered essential for acquiring 5HT fate [72]. Furthermore, FGF4 significantly reduces the expression of transcription factor PHOX2B, which is crucial for NA phenotype acquisition and, in turn, suppresses Fox2a gene expression [65,72]. Supporting the role of FGF4 in 5HT neuron differentiation, an increase in immunoreactivity towards the 5HT marker tryptophan hydroxylase 2 (TPH2) was observed following the differentiation procedure [72]. Subsequent to the acquisition of the 5HT phenotype, further neuronal maturation was achieved by supplementing the culture medium with growth factors, including BDNF, GDNF, TGFβ3, insulin growth factor-1 (IGF-1), and DAPT [65,72,73,89,102].

### 4.6. Cholinergic Differentiation

**Figure 1:**
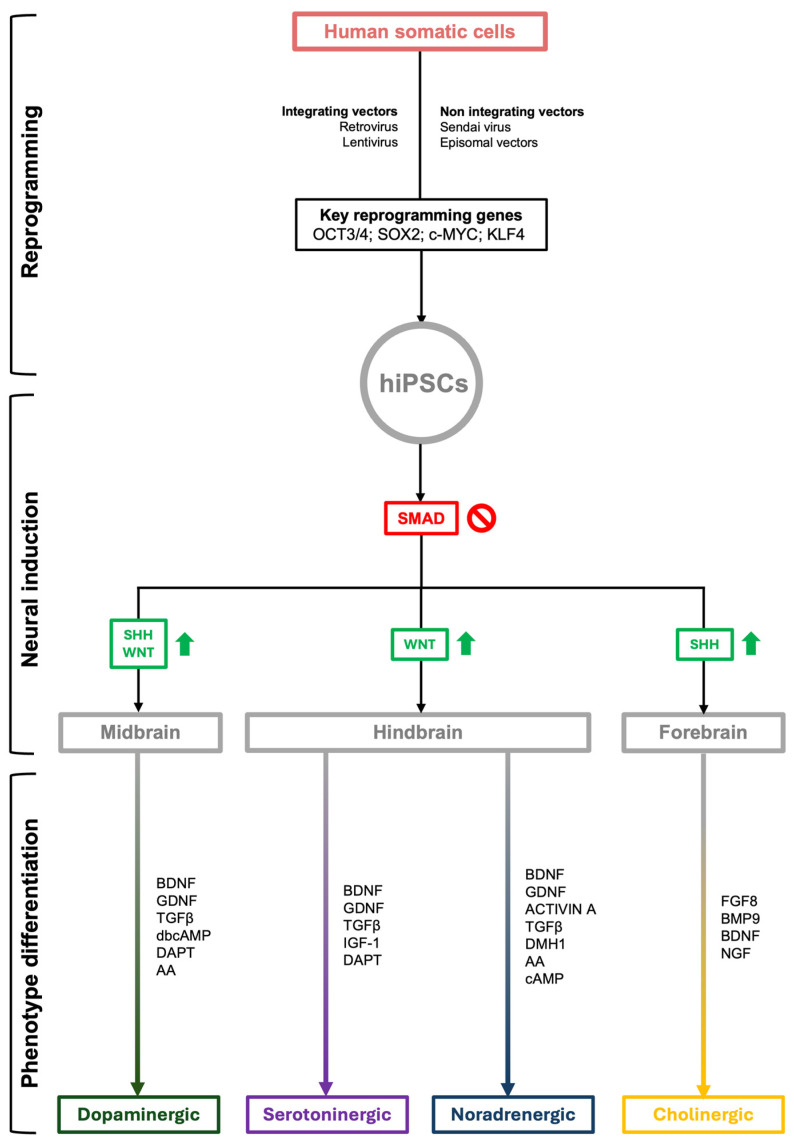
A schematic synthesis of NSS iPCS differentiation protocols. Starting from human somatic cells, either fibroblast or PBMC, a quite standardized reprogramming protocol is applied. Neural induction is obtained by inhibiting SMAD signaling and alternatively enhancing SHH and/or WNT pathways. Specific NSS phenotypes are then developed using standardized approaches, requiring specific growth factors, which are listed sequentially next to each arrow that connects the neural phenotype to the NSS phenotype. Abbreviations. AA—ascorbic acid; BDNF—brain-derived neurotrophic factor; BMP9—bone morphogenetic protein 9; c-MYC—myelocytomatosis oncogene; DAPT—N-[N-(3,5-difluorophenyl acetyl)-L-alanyl]-S-phenylglycine t-butyl ester; dbcAMP—dibutyryl cyclic AMP; FGF8—fibroblastic growth factor 8; GDNF—glial-derived neurotrophic factor; IGF-1—insulin growth factor 1; KLF4—Kruppel-like factor 4; NGF—nerve growth factor; OCT3/4—transcription factor POU domain class 5 transcription factor 1 (OCT3/4); SHH—sonic hedgehog; SOX2—sex-determining region Y-box2; TGFβ—transforming growth factor β.

Only one paper describing the generation of Ach neurons with basal forebrain identity from induced pluripotent stem cells [105] was included in this report. Following the generation of neural precursors using the SMAD inhibition approach and their expansion using fibroblastic growth factor 2 (FGF2), SHH was used to induce ventralization of neural progenitors. To promote telencephalic identity and Ach differentiation, the culture medium was supplemented with FGF-8 and BMP9, respectively. Lastly, during Ach maturation, BDNF and NGF were supplemented in culture medium until full neuronal development. See Figure 1 for a synthetic schematization of all the protocols described.

## 5. Experimental Findings and Insight into NDDs’ Pathophysiology 

In this section (and the following one), we describe the findings obtained from iPSC-derived models of NSSs. As a general premise, it should be noted that there is a significant disproportion among the selected studies in terms of neuronal phenotype analyzed, with nearly all of those focusing on the evaluation of the DA system in the context of PD and parkinsonisms, and very few, if any, concerning the other NSSs.

### 5.1. Dopaminergic IPSC Models

Many studies have been performed on iPSC models of midbrain DA neurons, revealing new elements that may be involved in the cellular pathogenesis of PD, thanks to the extensive manipulability of this model [125]. Interestingly, iPSCs obtained from PD patients’ samples in these studies showed morphological alterations consistent with PD pathology, such as alpha-synuclein (alpha-syn) accumulation, neurite arborization disruption, and mitochondrial abnormalities [79,126,127,128]. In 2012, Sanchez-Danes and colleagues obtained DA iPSCs from both idiopathic and genetic [bearing leucine-rich repeat kinase 2 (*LRRK2*) mutation] PD patients, [79]. They found that, compared with those of controls, DA neurons from patients showed reduced neurite arborization associated with autophagic pathway impairment [79]. These findings were confirmed by Ren and colleagues in a model obtained from *PARK2*-mutated PD patients, where neurite arborization disruption was linked to microtubular instability [126], a relation further confirmed in another iPSC study [127]. When cultured for long enough, DA iPSCs of patients start to accumulate intracellular alfa-syn, which is also released extracellularly [75,79,101,128,129]. According to Diao and colleagues, these might resemble Lewy body formation in case of *SNCA* triplication [101]. Zambon and colleagues found that alfa-syn pathology might be associated with the impairment of mitochondrial disfunction, as observed through the transcriptomic analysis of DA iPSCs obtained from *SNCA* patients [128]. Similar conclusions were drawn in another study on *PARK2*-mutated DA iPSCs, where the authors found impairment of mitochondria, and associated it with the disruption of their biogenesis [130]. 

DA iPSC models also allow for the dissection of cellular pathogenic mechanisms through which PD-related genetic mutations might cause the onset of the disease. The heterozygous mutation in the glucocerebrosidase gene (*GBA*) is the most significant genetic risk factor for PD and has been extensively studied using iPSC models. In 2016, Fernandes and colleagues developed DA iPSCs from donor patients and found that this mutation led to the misprocessing of beta-glucocerebrosidase within the endoplasmic reticulum, causing its accumulation and the disruption of autophagic pathways, eventually impairing DA synthesis and leading to alpha-syn accumulation [129]. Indeed, in another *GBA* iPSC study, Mazzulli et al. pharmacologically restored the activity of glucocerebrosidase, and showed, in parallel, the reduction of alfa-syn accumulation, the restoration of lysosomal activity, and the enhancement of DA neuron activity [131]. Previously, in 2015, Woodard et al. studied twins carrying a heterozygous mutation of the *GBA*, who were clinically discordant for the disease (one of the brothers was affected, while the other was not) [75]. They found that, while the iPSCs obtained from both twins showed lower levels of GBA activity, DA production, and accumulation of alfa-syn, the ones originating from fibroblasts of the affected brother showed an even lower level of DA synthesis and higher monoamine oxidase B (MAO-B) activity. This highlights how other factors might influence disease development [75], an observation inferred in other studies [132], and cases of different causes of mutation [133].

Mutations in the *LRRK2* gene are the most common cause of genetic PD. In DA iPSCs developed from affected patients, Lopez de Maturana and colleagues found that the abnormal function of this protein causes an alteration in alpha-syn homeostasis, promoting its intracellular accumulation, and it is associated with the impairment of the NF-κB pathway, leading to abnormal activation of neuroinflammation [134]. The role of the latter link was highlighted by Schmidt and colleagues, who found that microglia-derived factors are key in the development of DA iPSCs, suggesting a key neuroprotective effect on DA neurons [135]. In another study, the authors found that *LRRK2* mutation might be associated with the disruption of calcium homeostasis, impairing cellular metabolisms and exacerbating metabolic stress [136]. This very same pathway might also be recruited in cases of the PD-related mutation of *PINK1*, a gene coding for a serine/threonine kinase involved in mitochondrial activity, which was found to upregulate *LRRK2* activity in DA iPSCs [137]. In PD patients bearing the mutation for *PARK2*, a gene which encodes for E3 ubiquitin ligase parkin, the development of DA iPSCs showed the occurrence of the upregulation of the enzyme catechol-O-methyltransferase (COMT) together with the demethylation of its gene [138], shedding light on an otherwise unclear causative association [139].

Another interesting topic in which iPSCs have been contributing is the association between nicotine assumption and PD development. DA iPSCs express a nicotinic receptor [140], which promotes structural plasticity, increasing the number and the length of DA neuron dendrites and their somata size [141]. Forming a complex with a DA receptor, nicotinic ones exert neuroprotective effects on DA neurons, preventing the accumulation of alfa-syn occurring in cases of cellular distress [69,142] and promoting the transcription of neuronal growth factors [143]. As proof of concept, iPSCs obtained from *LRRK2* patients showed abnormal membrane localization of these receptors, whose neuroprotective function was impaired [144]. This line of research, entirely developed using DA iPSC models, might open a new therapeutic perspective for PD. 

Finally, some authors have explored the diagnostic utility of DA iPSC, as transcriptomic analysis performed in this model clearly identified gene and protein networks that can be affected and detected in PD patients [145]. In 2022, Ren and colleagues published a paper in which they reported the results of detailed molecular analysis performed on DA iPSCs obtained from healthy control and PD patients, the latter divided based on the occurrence of resting tremors [146]. They detected significant differences in terms of genetic expression, metabolites, and oxidative stress that allowed them not only to distinguish between patients and controls but also between patients with or without resting tremors, opening a possible scenario for a whole-informative diagnostic test [146]. 

### 5.2. Other NSS iPSC Models

For the sake of honesty, we did not find any published papers specifically regarding non-DA NSS iPSC in NDDs. However, the two studies published by Vadodaria and colleagues in 2019, using 5HT iPSC models [147,148], are worth mentioning. In those papers, the authors reported the results of their analysis on major depressive disorder patients, finding an abnormal response to 5HT in forebrain neurons [148] and morphological and developmental alterations in the neurites of 5HT iPSCs [147] when compared with healthy controls. Although included patients were not properly affected by NDDs, depression and mood disturbances are quite common prodromal symptoms in this group of diseases [149]. Thus, these might represent a further potential utility of iPSCs in the study of prodromal stages of NDDs.

As far as we know, there are no published articles yet considering NA and Ach iPSC models of NDDs.

## 6. Drug Testing and Therapy Development

iPSC models have also been used to open new therapeutic perspectives in NDDs. In this context, two main approaches have been pursued. On the one hand, iPSCs have been applied as in vitro platforms to screen pharmacological compounds and to test gene editing approaches; on the other, the same obtained iPSCs represent pharmacological intervention as in vivo transplantations have been attempted in order to restore the reproduced NSSs disrupted in NDDs.

### 6.1. In Vitro Evaluation of Drug Safety and Pharmacodynamics

As said, iPSCs can be reprogrammed to achieve the morphological and biochemical characteristics of brain NSS neurons (Section 4 and Section 5). Thus, they can be used in vitro assays to test drug efficacy and safety. DA iPSCs are sensitive to chemical compounds classically used to induce cellular stress and damage, underlining the reliability of this cellular model [98,150,151]. Some molecules have already been tested on these cells, with promising results [106,152,153,154,155,156]. In particular, ketamine and its derivate (2R,6R)-hydroxynorketamine have been shown to promote neurite growth and neural plasticity in DA iPSC models, with minimal or no adverse effects on cultured neurons [106,152,153], a beneficial effect that was identified also for the combination of L-acetylcarnitine and L-methylfolate [155]. In SNCA-mutated DA iPSCs, felodipine, a calcium antagonist, was found to ameliorate stress signatures, such as autophagy disruption and metabolic impairment [156]. Phorbol esters were tested in DA iPSCs obtained from genetic PD patients and they caused a reduction in alfa-syn pathology by acting as a lysosomal function activator [154]. Similar effects were interestingly observed by stimulating the nicotinic receptors of DA cells, as already reported in the previous section [141,142].

Finally, it is worth mentioning that iPSCs might also be used to evaluate the effectiveness of gene editing in treating genetic diseases. In DA iPSCs, the genetic manipulation through CRISPR-Cas9 of the progenitor can induce the occurrence of PD pathology [157,158], and the same cellular model can be applied to verify the utility of antisense oligonucleotide in silencing causative mutated genes [159].

### 6.2. In Vivo Transplantation of iPSCs

As expected, all the studies on the opportunity to transplant iPSCs in the human brain have been performed in PD models, as, in this disease, there is a specific degeneration of SNpc DA neurons, and the pharmacological replacement of DA release through L-DOPA administration represents the current standard therapy [160]. Thus, restoring the population of DA neurons would constitute a major achievement in PD treatment. Indeed, many preclinical studies have been performed, in which human-derived iPSCs were transplanted into the SNpc of mice with experimentally induced PD and showed promising results [67,88,92,104,161,162,163,164,165,166,167]. In fact, transplanted human DA iPSCs usually survived in the host animal [92,104,161,162,163,164,166] and showed neurite growth [88,92,163] and DA innervation of appropriate brain targets [67]. Furthermore, treated animals showed improvement in motor symptoms and behavior, showing that transplanted iPSCs not only can survive and integrate in the host brain but can also restore the original physiological functions of degenerated DA neurons [67,88,92]. Some factors seem to the influence success rate and quality of transplanted neurons, such as the age of the precursor cells or of the same iPSCs [164,165,167] and the vitality of support neurons [161]. Moreover, it should be noted that producing iPSCs from patients might lead to obtaining more fragile DA neurons; in one study, iPSCs were cultured from genetic PD patients and these developed PD pathology once transplanted in the SNpc of the animal model [166]. As we reported above (Section 5.1), DA iPSCs obtained from idiopathic PD patients showed pathological alterations compared with controls, and this might hinder the usefulness of autologous transplantation without any further editing. However, in 2020, this same approach was experimented in a PD patient; DA iPSCs were obtained from skin fibroblasts and implanted bilaterally in the putamen of the subject. The graft survived and the clinical outcome of the patient improved, with the reduction or stabilization of motor symptoms [168].

## 7. Discussion and Future Perspectives

In this review, we provide a detailed overview of published studies in which the authors either developed specific protocols to obtain iPSC models of NSSs or utilized these models in the context of NDD studies. iPSCs offer a promising opportunity to directly study human neuron cytotypes, whose features and functions would otherwise be extremely difficult to analyze. Our aim was to identify which specific systems these in vitro models are best suited to represent and to describe the state of the art regarding how reliably they can replicate NSS neurons. Indeed, we found that all of the main NSSs can be “replicated” through iPSC modeling, with refined protocols available for DA SNpc, NA LC, 5HT DRN, and Ach forebrain neurons.

On the other hand, our literature search revealed a clear trend on the use of these models in NDD research, with most published articles focusing on the DA NSS, and only a few papers reporting data about the 5HT, Ach, and NA systems. Studies on DA iPSCs have led to the significant advancement of our understanding of PD pathophysiology, through metabolic, biochemical, and genetic analyses. Moreover, they have led to the first human autologous graft [168], elevating the role of DA iPSCs from that of mere experimental models to the source of potential therapeutic candidates [169]. 

It might also be supposed that the literature regarding those topics will significantly increase very soon. For instance, in 2023, Tao and colleagues developed a refined protocol to obtain well-differentiated NA iPSCs with characteristics specific to LC-NA neurons [99]. This achievement offers an opportunity to thoroughly study the metabolic and cytological features of a system that is often and early involved in NDDs, such as PD [46] and AD [10]. In particular, in AD, the LC is the first brain structure to show tau-related pathological alterations [44], and many hypotheses have been proposed to explain its sensitivity to degenerative damage [170], including oxidative stress related to catecholamine synthesis and degradation [171], predisposition to tau protein hyperphosphorylation [172,173,174], and secondary effects of systemic inflammation and xenobiotics [175,176,177,178]. All these hypotheses could be tested in NA iPSCs carrying the genetic landscape of patients affected by either sporadic or genetic AD, shedding light on an unclear yet probably crucial step in the pathogenesis of AD.

Nonetheless, we must acknowledge the current lack of information on non-DA iPSC models for NDD research as a limitation of our review. Their pleiotropic roles in NDDs and less refined differentiation protocols may partially explain why only a few studies have focused on other NSSs. However, this limits our ability to assess the potential utility of these in vitro models beyond thae specific case of the DA system in PD. Further extensive studies are required to fully uncover the potential applications of these iPSC models in other NDDs.

One of the most interesting findings is that iPSCs obtained from donors affected by NDDs and reprogrammed show pathological features not only in cases of genetic forms but also in sporadic ones, as reported in [79,145,146]. Some relevant implications are worth noting. From the pathophysiological point of view, it highlights the crucial contribution of polygenetic and epigenetic factors to the occurrence of NDDs, as the iPSCs obtained from sporadic PD patienats showed pathological alterations similar to those developed from the genetic PD ones, even in the absence of any external influence. Methodologically, this characteristic bears a dual meaning. On the one hand, it reinforces the reliability of iPSCs as experimental models for sporadic forms of NDDs, where no clear genetic mutations are present, and a heterogeneous mixture of genetic and environmental factors likely triggers the pathogenic process. On the other hand, it emphasizes the importance of checking for the occurrence of other pathological or para-physiological factors related to the genetic and epigenetic landscape of donor patients. Mutations with low penetrance, even with no or minimal clinical manifestations, might still affect the biochemistry and metabolism of iPSCs, impairing the reliability and reproducibility of the collected results. As these models are further applied and developed, it will become increasingly important to accurately define and consider their genetic and epigenetic backgrounds.

However, extending this topic, it is conceivable that, in the near future, iPSCs might indeed be used as diagnostic tools. They will allow for the easy definition of the genomic and proteomic profiles of affected patients, identifying specific disease subtypes with unprecedented accuracy. Taking this concept to its extreme, one might even anticipate using iPSCs in the future to screen for the propensity to develop NDDs in otherwise asymptomatic individuals. Furthermore, the effects of drugs could be tested directly on target neurons, offering the opportunity to choose therapeutic strategies that best fit the characteristics of each patient, aligning with the personalized medicine approach.

Such future developments will inevitably require reducing or, at the very least, controlling possible variability. This could involve addressing methodological inconsistencies by defining strict and standardized protocols for iPSC differentiation. Indeed, our literature search revealed a notable degree of variability in current approaches to NSS phenotype differentiation across different research groups. Achieving this step will be mandatory to ensure the accuracy and reproducibility necessary for useful diagnostic tools.

In conclusion, NSS iPSCs are a useful and promising in vitro model for the study of NDDs. They have been extensively used in PD research, providing valuable and otherwise elusive findings. Extending their application to other NSSs and NDDs might be an excellent strategy to increase our understanding of the many roles of subcortical structures in this class of neurological disorders.

## Figures and Tables

**Table 1:** The main neuromodulatory subcortical nuclei.

Anatomical Definition	Histochemical Classification (Neurotransmitter)	CNS Targets	Physiological Effects
Dorsal Raphe Nucleus (DRN)	B8–B9 Serotonin (5HT)	Brainstem, hypothalamus, amygdala, hippocampus, cortex	Wake-promoting nucleus; pain and vegetative function regulation; mood and behavior modulation.
Locus Coeruleus (LC)	A6 Noradrenaline (NA)	Virtually the whole CNS	Wake-promoting nucleus; attention focusing and shifting; learning and memory promotion.
Substantia Nigra pars compacta (SNpc)	A9 Dopamine (DA)	Basal ganglia, thalamus, motor cortex	Movement regulation; planning and execution
Ventral Tegmental Area (VTA)	A10 Dopamine (DA)	Amygdala, hippocampus, nucleus accumbens, frontal cortex	Reward process and reinforced learning promotion; executive functions regulations.
Pedunculopontine Nucleus (PPN) and Lateral Tegmental Nucleus (LTN)	Ch5 and Ch6 Acetylcholine (Ach)	Other brainstem nuclei, hypothalamus	Wake-promoting nuclei
Nucleus Basalis of Meynert (NBM), Medial Septum Nucleus (MSN), horizontal and vertical limbs of the diagonal band of Broca (hDBB and vDBB)	Ch1, Ch2, Ch3 and Ch4 Acetylcholine (Ach)	Isocortex (NBM), hippocampus (MSN and vDBB), and olfactory cortex (hDBB)	Cortical activation and desynchronization; learning and memory promotion.

Legend to table. Ach—acetylcholine; CNS—central nervous system; DA—dopamine; DRN—dorsal raphe nucleus; hDBB—horizontal limb of the diagonal band of Broca; LC—locus coeruleus; LTN—lateral tegmental nucleus; MSN—medial septum nucleus; NA—noradrenaline; NBM—nucleus basalis of Meynert; PPN—pedunculopontine nucleus; SNpc—substantia nigra pars compacta; vDBB—vertical limb of the diagonal band of Broca; VTA—ventral tegmental area; 5HT—serotonin.

## Abbreviations

AA—ascorbic acid; Ach—acetylcholine; AD—Alzheimer’s disease; Alpha-syn—alpha synuclein; BDNF—brain-derived neurotrophic factor; BMP—bone morphogenetic protein; c-MYC—myelocytomatosis oncogene; CNS—central nervous system; COMT—catechol-O-methyltransferase; DA—dopamine; dbcAMP—dibutyryl cyclic AMP; DAPT—N-[N-(3,5-difluorophenyl acetyl)-L-alanyl]-S-phenylglycine t-butyl ester; DRN—dorsal raphe nucleus; FGF—fibroblast growth factor; hDBB—horizontal limb of the diagonal band of Broca; GBA—glucocerebrosidase; GDNF—glial-derived neurotrophic factor; IGF—insulin growth factor; iPSCs—induced pluripotent stem cells; KLF4—Kruppel-like factor 4; LC—locus coeruleus; LHA—lateral hypothalamic area; LRRK2—leucine-rich repeat kinase 2; LTN—lateral tegmental nucleus; MAO-B—monoamine oxidase B; MSN—medial septum nucleus; NA—noradrenaline; NBM—nucleus basalis of Meynert; NDD—neurodegenerative disorder; NSS—neuromodulatory subcortical system; OCT3/4—transcription factors POU domain class 5 transcription factor 1; PBMC—peripheral blood mononuclear cell; PD—Parkinson’s disease; PPN—pedunculopontine nucleus; SHH—sonic hedgehog; SNpc—substantia nigra pars compacta; SOX2—sex-determining region Y-box2; TGF- β—transforming growth factor β; vDBB—vertical limb of the diagonal band of Broca; VTA—ventral tegmental area; 5HT—serotonin.
